# A General and Efficient Strategy for Gene Delivery Based on Tea Polyphenols Intercalation and Self‐Polymerization

**DOI:** 10.1002/advs.202302620

**Published:** 2023-06-22

**Authors:** Hao Chen, Lina Guo, Jinsong Ding, Wenhu Zhou, Yan Qi

**Affiliations:** ^1^ Department of Pathology Zhanjiang Central Hospital Guangdong Medical University Zhanjiang Guangdong 524000 China; ^2^ Department of Pathology Shihezi University School of Medicine Shihezi Xinjiang 832002 China; ^3^ Department of Pharmaceutics Xiangya School of Pharmaceutical Sciences Central South University Changsha Hunan 410013 China

**Keywords:** acute hepatitis, gene delivery, nanoparticles, self‐polymerization, targeting

## Abstract

Gene therapy that employs therapeutic nucleic acids to modulate gene expression has shown great promise for diseases therapy, and its clinical application relies on the development of effective gene vector. Herein a novel gene delivery strategy by just using natural polyphenol (‐)‐epigallocatechin‐3‐O‐gallate (EGCG) as raw material is reported. EGCG first intercalates into nucleic acids to yield a complex, which then oxidizes and self‐polymerizes to form tea polyphenols nanoparticles (TPNs) for effective nucleic acids encapsulation. This is a general method to load any types of nucleic acids with single or double strands and short or long sequences. Such TPNs‐based vector achieves comparable gene loading capacity to commonly used cationic materials, but showing lower cytotoxicity. TPNs can effectively penetrate inside cells, escape from endo/lysosomes, and release nucleic acids in response to intracellular glutathione to exert biological functions. To demonstrate the in vivo application, an anti‐caspase‐3 small interfering ribonucleic acid is loaded into TPNs to treat concanavalin A‐induced acute hepatitis, and excellent therapeutic efficacy is obtained in combination with the intrinsic activities of TPNs vector. This work provides a simple, versatile, and cost‐effective gene delivery strategy. Given the biocompatibility and intrinsic biofunctions, this TPNs‐based gene vector holds great potential to treat various diseases.

## Graphical abstract

This work provides a simple, versatile, and cost‐effective gene delivery strategy based on natural polyphenols intercalation and self‐polymerization, which can load any types of nuclei acids with high efficiency and possesses much lower cytotoxicity than commonly used cationic materials. The intrinsic radical scavenging and anti‐inflammatory activities of the vector can also synergize the gene therapy to treat inflammatory diseases.

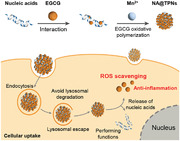

## 1 Introduction

Gene therapy has been recognized as a promising strategy to treat intractable diseases, which employs genetic materials to manipulate gene expression for therapeutic purposes.^[^ ^1^, ^2^ ^]^ Currently, various types of therapeutic nucleic acids, such as antisense oligonucleotide (ASO),^[^ ^3^ ^]^ small interfering ribonucleic acid (siRNA),^[^ ^4^ ^]^ messenger RNA (mRNA),^[^ ^5^ ^]^ plasmid DNA (pDNA),^[^ ^6^ ^]^ and CRISPR^[^ ^7^ ^]^ complexes, have been developed as gene therapy, and they function by correcting the genetic defect in the chromosome, supplying the lacking components, or suppressing the production of undesired protein. Since these nucleic acids are only active at specific cellular compartments where they are required, a common task of gene therapy is the development of effective gene vectors to deliver exogenous nucleic acids, and several challenges should be carefully considered for in vivo application.^[^ ^8^, ^9^, ^10^, ^11^ ^]^ First, nucleic acids, especially the RNAs, are relatively labile biomolecules that are prone to biological degradation during in vivo circulation. Second, they are highly negatively charged polymers that cannot permeate through the lipid bilayer of cell membrane. Even after being swallowed by cells, successful endo/lysosome escape is also required for their intracellular functions.^[^ ^8^ ^]^ Therefore, a successful delivery system should be biocompatible, and must protect nucleic acids from premature degradation, effectively transport across cellular membrane, and deliver to subcellular site of action.

Up to now, a broad diversity of viral and nonviral delivery systems have been used to deliver therapeutic nucleic acids for gene therapy.^[^ ^12^, ^13^ ^]^ While viralvectors possess superiority of high stability and excellent transfection efficiency, the intrinsic limitations such as immunogenicity, toxicity, and high cost severely hinder their widespread biomedical applications.^[^ ^14^ ^]^ Alternatively, the nonviral vectors have attracted significant attention in recent years, in which cationic lipids and polymers are most extensively studied. These vectors could package nucleic acids via electrostatic attraction, protect nucleic acids from nuclease degradation, and transfect into cells with high efficiency.^[^ ^15^ ^]^ However, these positively charged vectors could adsorb large amounts of serum proteins during circulation to accelerate in vivo clearance, and their inherent toxicities induce strong side‐effects. To avoid these limitations, non‐positively charged vectors were also developed by conjugating nucleic acids into nanoparticles, which however requires complicated synthesis procedure, and the transfection efficiency is strongly affected by the size, shape, and surface environment of the nanoparticles.^[^ ^16^ ^]^ Therefore, the development of simple, biosafe, efficient, yet cost‐effective nucleic acids vector is still highly desirable for gene therapy.

Several small molecular compounds that can intercalate into polynucleotide bases have been reported to promote gene delivery by virtue of their capability to condense nucleic acids.^[^ ^17^, ^18^ ^]^ For example, Cheng and coworkers reported the use of a natural polyphenol (‐)‐epigallocatechin‐3‐O‐gallate (EGCG) as intercalation agent to facilitate siRNA delivery.^[^ ^19^ ^]^ EGCG could interact with nucleic acids via terminal base‐pair stacking, intercalation or groove binding, and the large catechins from EGCG endow good stability of the EGCG‐nucleic acids complexes.^[^ ^17^, ^20^, ^21^ ^]^ Such entropy‐driven complexation of siRNA with EGCG yielded a negatively charged core, which allowed cationic polymers coating to form uniformed nanoparticles. While this pre‐complexation strategy could significantly improve the siRNA delivery efficiency, cationic vectors were still required for stable delivery.

**Scheme 1:**
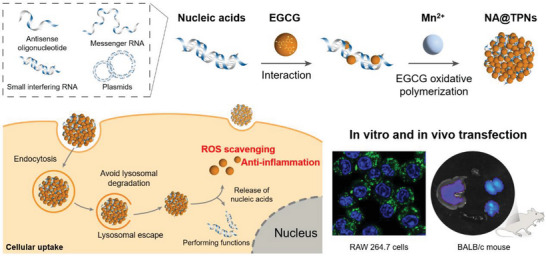
Schematic illustration the construction of general gene vector to deliver various types of nucleic acids for in vitro and in vivo transfection by using EGCG as raw material, which is achieved via its intercalation and self‐polymerization.

Interestingly, polyphenols could form nanoparticles via autoxidation and polymerization, and the widely studied example is the use of dopamine to synthesize polydopamine.^[^ ^22^, ^23^, ^24^ ^]^ We recently found that EGCG could oxidize and polymerize to tea polyphenols nanoparticles (TPNs) under mild condition with the catalysis of Mn^2+^.^[^ ^25^ ^]^ These self‐assembled nanoparticles were highly stable and biocompatible, which have found application for disease treatment. Inspired by this fact, herein we report a novel type of gene delivery strategy by using EGCG as raw material (**Scheme** **1**). EGCG pre‐intercalates into nucleic acids, followed by adding Mn^2+^ to catalyze EGCG oxidation and self‐polymerization to form nanoparticles, through which nucleic acids were stably encapsulated. It is a mild and simple preparation process under ambient condition with only 2 h reaction. The versatility of such delivery system has been confirmed to load various types of therapeutic nucleic acids, including ASO, siRNA, mRNA, and pDNA, and its efficiency has also been demonstrated both in vitro and in vivo, providing a general and non‐cationic gene vector with minimal toxicity.

## 2 Experimental Section

### 2.1 Materials

The ASO, siRNA, FAM‐ or Cy5.5‐labeled counterpart (Random Sequence, Non‐functional), and anti–TNF–α siRNA (siTNF‐*α*) were bought from Bioegene (Shanghai, China). The anti–caspase–3 siRNA (sicaspase‐3) was purchased from Genepharma (Shanghai, China). ARCA‐EGFP mRNA was purchased from APExBIO (Houston, USA). The pDNA encoding EGFP was extracted from *Escherichia coli* using a plasmid extraction kit (Omega, Connecticut, USA). EGCG was bought from Huagao Biological Products (Chengdu, China). Manganese chloride tetrahydrate (MnCl_2_·4H_2_O) and polyethyleneimine (PEI) were bought from Aladdin (Shanghai, China). 1,2–dioleoyl–3–trimethylammonium–propane (DOTAP) was purchased from Chemical Book (Shanghai, China). Fetal bovine serum (FBS) was purchased from BI (Israel). Lipopolysaccharide (LPS), DCFH‐DA and concanavalin A (Con A) were purchased from Sigma (St Louis, MO, USA). The primers for RT‐PCR experiments (*β*‐actin, TNF‐*α*, IL‐1*β*, IL‐6, caspase‐3) were synthesized by Sangon Biotech (Shanghai, China) and the sequence is shown in Table S1, Supporting Information. Hoechst 33342 was purchased from Thermo Fisher Scientific (Massachusetts, USA). The kits for alanine aminotransferase (ALT), aspartate aminotransferase (AST), creatinine (Cre), and blood urea nitrogen (BUN) were purchased from Jiancheng Institute of Biological Engineering (Nanjing, China). The kits for malondialdehyde (MDA) and hydrogen peroxide (H_2_O_2_) were purchased from Beyotime Biotechnology (Shanghai, China). The ELISA kits for TNF‐*α*, IL‐1*β*, and IL‐6 were purchased from Meimian Industrial (Jiangsu, China).

### 2.2 Preparation of Nucleic Acids@TPNs (NA@TPNs)

TPNs were prepared by EGCG polymerization at alkaline condition under the catalysis of Mn^2+^. EGCG was first added into HEPES buffer (10 mm, pH 8.0) and stirred for 5 min. MnCl_2_ was then added dropwise, followed by stirring at 37 °C for 1 h. The final concentration of EGCG and MnCl_2_ were 2.5 and 2 mm, respectively. The nanoparticles were collected by centrifugation at 16 000 rpm for 15 min, washed twice with HEPES buffer (10 mm, pH 7.4), sonicated to be dispersed and stored at 4 °C for further use.

To prepare NA@TPNs, the nucleic acids were added to the HEPES buffer at the desired concentration along with EGCG for premix. After 30 min of stirring, MnCl_2_ was added and the reaction time was extended to 2 h. The NA@TPNs were then collected and redispersed as described above.

### 2.3 Characterization of NA@TPNs

Fluorescence quenching experiments were used to demonstrate the potential of TPNs as nucleic acid carriers. The supernatant was collected and examined by 3% agarose gel electrophoresis to determine the nucleic acid loading capacity of TPNs. The ZetaSizer Nano ZS (Malvern Instruments, UK) was used to examine the particle size, polydispersity index (PDI) and *ζ* potential of TPNs and NA@TPNs. Transmission electron microscope (TEM) (Titan G2‐F20, FEI, USA) was used to obtain morphological and elemental profiles of the nanoparticles. The NA release from NA@TPNs was investigated by collecting the supernatants at different timepoints and performing gel electrophoresis. The TPNs and NA@TPNs were stored at 4 °C, and the size was measured every 2 days within 15 days to investigate the storage stability.

### 2.4 Serum Stability Assay

The naked FAM‐siRNA or FAM‐siRNA@TPNs were incubated with 10% FBS at 37 °C for 0, 2, 4, or 8 h. The samples were then treated with glutathione (GSH) to release the encapsulated siRNA. Afterward, gel electrophoresis was performed to detect the cleavage bands. The degradation rate of siRNA was quantified by ImageJ.

### 2.5 Preparation of NA@PEI and NA@DOTAP

An enzyme‐free aqueous solution of 2 mg mL^−1^ PEI was prepared, and an appropriate amount of PEI was stirred with siRNA in 1 mL HEPES buffer (pH 7.4 10 mm) for 30 min at room temperature, followed by centrifugation at 16 000 rpm for 15 min to obtain NA@PEI nanoparticles.

DOTAP was dissolved in chloroform and then evaporated in a vacuum rotary evaporator to obtain a translucent film. The film was then hydrated by sonication to obtain DOTAP liposomes at a concentration of 2 mg mL^−1^. After incubation with FAM‐siRNA for 30 min at room temperature, the nanoparticles were centrifuged at 16 000 rpm for 15 min to obtain NA@DOTAP nanoparticles.

### 2.6 Protein Corona Formation

The prepared NA@TPNs, NA@PEI, and NA@DOTAP were incubated with 10% FBS at 37 °C for 1 h. Afterward, the samples were centrifuged at 16 000 rpm for 15 min, with the supernatant and precipitates collected, respectively. The concentration of unadsorbed protein in the supernatant was determined by BCA protein kit. The precipitates were redispersed, denatured, and separated by gel electrophoresis, followed by staining with Coomassie brilliant blue.

### 2.7 Cell Culture

The murine macrophages RAW264.7 cells were obtained from Xiangya Cell center (Changsha, China) and cultured in RPMI‐1640 complete medium (supplemented with 10% FBS and 1% streptomycin/penicillin solution) at 37 °C in a 5% CO_2_ atmosphere.

### 2.8 Cell Viability Study

#### 2.8.1 MTT Assay

The RAW264.7 cells were seeded in 96‐well plates and cultured overnight. The different concentrations of TPNs, siRNA@TPNs, NA@PEI, and NA@DOTAP were added for incubation. After 24 h, the medium was replaced with MTT (0.5 mg mL^−1^) and further incubated for 4 h. Then, the medium was discarded and 100 µL DMSO was added to dissolve the formazan. The absorbance at 490 nm was measured by a microplate reader (Infinite M200 PRO, TECAN, Austria).

#### 2.8.2 Live/Dead Staining

The RAW264.7 cells were seeded in 24‐well plates and cultured overnight. The TPNs or siRNA@TPNs (siRNA equivalent concentration 400 nm) were added for incubation. After 24 h, calcein‐AM and PI were added and incubated at 37 °C for 30 min to stain the live and dead cells, respectively. The cells were then imaged by fluorescence microscope.

### 2.9 In Vitro Transfection

#### 2.9.1 ASO/siRNA

The RAW264.7 cells were seeded in 24‐well plates and cultured overnight. The ASO@TPNs or siRNA@TPNs (prepared with ASO/siRNA labeled by FAM) were added for 2, 4, or 8 h incubation, with naked ASO/siRNA (equivalent concentration 400 nm) incubated for 8 h as control. The cells were then collected and fluorescence intensity was measured by flow cytometry. In addition, the cells were treated with naked ASO/siRNA, ASO@TPNs, or siRNA@TPNs for 8 h, and then fixed with 4% paraformaldehyde for 10 min, followed by staining the cell nuclei with DAPI (1 µg mL^−1^). The cell internalization was observed by fluorescence microscope.

The RAW264.7 cells were seeded in 6‐well plates and stimulated with LPS (10 µg mL^−1^) for 24 h. The TPNs or siTNF‐*α*@TPNs (siRNA equivalent concentration 500 nm) were added for 24 h incubation. Then, total RNA was extracted by TRIzol and cDNA was synthesized by reverse transcription. The mRNA expression levels of TNF‐*α* were quantified by RT‐PCR (CFX‐Connect, BIO‐RAD, USA). In addition, RIPA lysis buffer was added to the cells post above treatments to extract the total protein, which was then quantified by BCA Protein Quantitation Kit. The samples were loaded and separated on sodium dodecyl sulfate‐polyacrylamide gel electrophoresis, followed by transferring to the PVDF membrane. Afterward, the membranes were blocked with 5% non‐fat milk for 1 h and then incubated with the primary antibody against TNF‐*α* at 4 °C overnight. The secondary antibodies were then added for 1 h incubation and the bands were visualized by ECL.

#### 2.9.2 mRNA/pDNA

The RAW264.7 cells were seeded in 24‐well plates and cultured overnight. The mRNA@TPNs or pDNA@TPNs (prepared with mRNA/pDNA encoding GFP) were added for 24, 36, or 48 h incubation, with naked mRNA/pDNA (equivalent concentration 2 µg mL^−1^) incubated for 48 h as control. The cells were then collected and fluorescence intensity was measured by flow cytometry. In addition, the cells were treated with naked mRNA/pDNA, mRNA@TPNs, or pDNA@TPNs for 36 h. The GFP expression was imaged by fluorescence microscope post fixation and cell nuclei staining as described above.

### 2.10 Endocytosis Pathways Study

The RAW264.7 cells were seeded in 24‐well plates and cultured overnight. The siRNA@TPNs (siRNA equivalent concentration 400 nm) prepared with FAM‐labeled siRNA were added post pre‐treating the cells with chlorpromazine (15 µg mL^−1^), nystatin (15 µg mL^−1^), colchicine (5 µg mL^−1^), or NaN_3_ (1 mg mL^−1^) for 1 h. After 8 h, the cells were collected and the fluorescence intensity was analyzed by flow cytometry.

### 2.11 Endosomal Escape

The RAW264.7 cells were seeded in confocal dishes and cultured overnight. The siRNA@TPNs (siRNA equivalent concentration 400 nm) prepared with FAM‐labeled siRNA were added for 2, 4, or 12 h incubation. Afterward, LysoTracker were added in the medium and incubated at 37 °C for 1 h to label lysosomes, followed by washing with PBS for three times. Then, the cells were fixed with 4% paraformaldehyde for 10 min and stained the cell nuclei with Hoechst 33342 (10 µg mL^−1^) for 5 min. The intracellular localization was observed by confocal laser scanning microscopy.

### 2.12 DPPH Scavenging

Different concentrations of TPNs (10, 25, 50, 100 µg mL^−1^, 50 µL) were mixed with DPPH ethanol solution (0.3 mM, 50 µL) and incubated in the dark. Absorbance at 517 nm was recorded every 5 min using a microplate reader and UV–visible spectra within 450–650 nm were recorded post 15 min.

### 2.13 NO Scavenging

To assess the ability to scavenge ·NO, ·NO was generated with sodium nitrate. Simultaneously, different concentrations of TPNs (10, 25, 50, and 100 µg mL^−1^) were added. After incubation for 30 min, Griess reagent was added for ·NO detection. The UV–vis spectra within 410–650 nm were recorded using a microplate reader.

### 2.14 ·OH Scavenging

To assess ·OH scavenging capacity, ·OH was generated by a Fenton‐like reaction with the Mn^2+^/H_2_O_2_ system. Simultaneously, different concentrations of TPNs (10, 25, 50, and 100 µg mL^−1^) and methylene blue (10 µg mL^−1^) were added. After incubation at 37 °C for 30 min, the UV–vis spectra within 400–800 nm were recorded using a microplate reader.

### 2.15 Intracellular RONS Scavenging Activity

The RAW264.7 cells were seeded in 24‐well plates and stimulated with LPS (10 µg mL^−1^) for 24 h. The TPNs or siRNA@TPNs (TPNs equivalent concentration 62.5 µg mL^−1^) were added for 24 h incubation. Afterward, various reactive oxygen and nitrogen species(RONS) probes including DCFH‐DA (10 µm) for general ROS, HPF (20 µm) for ·OH/ONOO^−^, DHE (5 µm) for ·O_2_^−^, and DAF‐FM DA (10 µm) for ·NO were added and incubated at 37 °C for 30 min. Then, the cells were observed by fluorescence microscope. In addition, the cells post the above treatments were collected and analyzed by flow cytometry.

### 2.16 Macrophage Repolarization

The RAW264.7 cells were seeded in 24‐well plates and stimulated with LPS (10 µg mL^−1^) for 24 h. The TPNs or siRNA@TPNs (TPNs equivalent concentration 62.5 µg mL^−1^) were added for 24 h incubation. Afterward, the cells were fixed with 4% paraformaldehyde for 10 min and then blocked with 5% BSA for 30 min. Then, fluorescence‐labeled antibodies against iNOS or CD206 were added for 1 h incubation, followed by washing with PBS for three times. The cells were imaged by fluorescence microscope after staining the cell nuclei with DAPI (1 µg mL^−1^). In addition, the cells post the above treatments were collected and analyzed by flow cytometry.

### 2.17 Anti‐Inflammatory Activity

The RAW264.7 cells were seeded in 6‐well plates and stimulated with LPS (10 µg mL^−1^) for 24 h. The TPNs (62.5 µg mL^−1^) were added for 24 h incubation. Afterward, total RNA was extracted and the mRNA expression levels of TNF‐*α*, IL‐1*β*, and IL‐6 were quantified by RT‐PCR (CFX‐Connect, BIO‐RAD, USA). In addition, the total protein of the cells post above treatments was extracted and the protein expression of TNF‐*α*, IL‐1*β*, and IL‐6 were examined by western blot as described above.

### 2.18 Animals

The 8‐week‐old male BALB/c mice were provided by Silaike Jingda Laboratory Animal Company (SJA, Hunan, China). They were fed in a sterile environment with an unlimited diet. All the protocols were approved by the Experimental Animal Ethics Committee of Zhanjiang Central Hospital with the assigned approval/accreditation number of ZJDY2022‐52.

### 2.19 Hemolysis Analysis

The blood of BALB/c mice was collected in anticoagulant tubes and centrifuged at 1000 rpm for 5 min. The erythrocytes were collected and washed with saline for several times until the supernatant became clear. Then, the supernatant was discarded and saline was added to obtain 2% erythrocyte suspension. Different concentrations of TPNs or siRNA@TPNs (10, 20, 40, 80, 160, and 320 µg mL^−1^) were added for 3 h incubation, with saline as negative control and H_2_O as positive control. Afterward, the state of the cell suspension was observed (no hemolysis, partial hemolysis, complete hemolysis, agglutination) and the absorbance of the supernatant at 541 nm was measured by microplate reader (Infinite M200 PRO, TECAN, Austria).

### 2.20 In Vivo Distribution

The mice were randomly divided into three groups and administered with PBS, naked Cy5.5‐siRNA, or Cy5.5‐siRNA@TPNs by intravenous injection (siRNA 1 mg kg^−1^, TPNs 12.5 mg kg^−1^). The major organs (heart, liver, spleen, lung, and kidneys) of the mice were harvested at 24 h post the injection and imaged using in vivo imaging system (PerkinElmer, USA).

### 2.21 Con A‐Induced Hepatitis and Treatment

Con A is a plant‐derived haemagglutinin. When injected at high doses it accumulated in the liver, activated the immune system, and induced T‐cell‐dependent liver damage. It was widely used to model viral hepatitis and autoimmune liver disease. The prophylactic treatment was the preferential treatment owing to the severity and rapid progression of the injury. In this study, TPNs or siRNA@TPNs were first injected intravenously (siRNA 1 mg kg^−1^, TPNs 12.5 mg kg^−1^) and Con A was administered through a tail vein injection at a dose of 20 mg kg^−1^ 36 h later.

### 2.22 Survival Analysis

The survival of mice post different treatments was observed and recorded during the next 48 h. The survival rate was calculated accordingly.

### 2.23 Histology Analysis

The livers of mice were harvested at 4 h post Con A administration, followed by photographing and weighing. Afterward, they were fixed in 4% formaldehyde, embedded in paraffin, sectioned, and H&E and TUNEL staining was performed. In addition, the sections were incubated with the primary antibody against caspase‐3 at 4 °C overnight and then fluorescence‐labeled secondary antibody for 1 h. The cell nuclei were stained with DAPI and the sections were imaged by a fluorescence microscope.

### 2.24 ALT/AST Quantification

The blood of mice was collected post treatment and the serum was separated by centrifugation. The serum levels of ALT and AST were measured by corresponding kits according to the instructions.

### 2.25 Antioxidative Activity In Vivo

The fresh liver samples of mice were homogenized in specific lysis buffer, followed by centrifugation to collect the supernatant. The H_2_O_2_ and MDA levels in these samples and the abovementioned serum samples were measured by corresponding kits as instructed. In addition, protein carbonyl content kits were used to measure the protein carbonyl content in the liver of mice post different treatment.

### 2.26 Anti‐Inflammatory Activity In Vivo

The fresh liver samples of mice were homogenized in PBS and the supernatant post centrifugation was collected. The levels of inflammatory cytokines including TNF‐*α*, IL‐1*β*, and IL‐6 in these supernatant as well as in serum were measured by corresponding ELISA kits.

### 2.27 Safety Evaluation

At the termination of the in vivo experiments, blood was collected from each group of mice and tested for BUN and Cre using blood biochemistry kits. The major organs were obtained and fixed in 4% formaldehyde for further H&E staining and analysis of pathological damage.

### 2.28 Statistical Analysis

All quantitative data were presented as mean ± standard deviation (SD). For each experiment, a minimum of three replicates was performed and the specific number of replicates per independent experiment was noted in the corresponding figure legends. GraphPad Prism 8 was used for graphing and statistical analysis. Student's *t*‐test, one‐way ANOVA, and two‐way ANOVA were used to compare various groups. The significance was defined as follows: *p < 0.05, **p < 0.01, ***p < 0.001, ****p < 0.0001.

## 3 Results and Discussions

### 3.1 TPNs as a Robust Gene Vector for siRNA Loading

**Figure 1:**
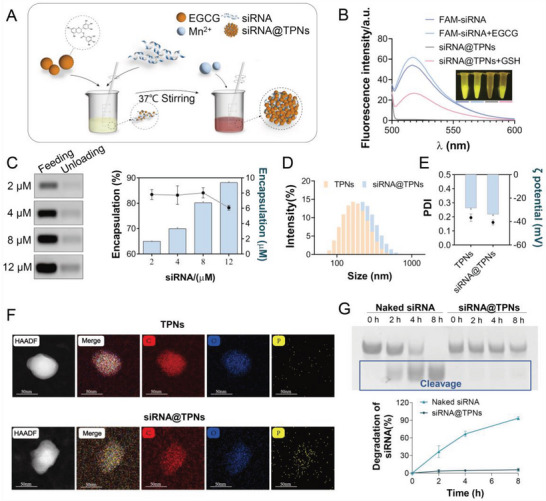
Preparation and characterizations of siRNA@TPNs. A) Schematic illustration of the process of siRNA@TPNs preparation. B) The fluorescence spectra and photographs of various samples. C) Gel images and intensity quantification to show siRNA loading capacity of TPNs at different feeding concentrations (*n* = 3). D) The size distribution, E) PDI and *ζ* potential, and F) Elemental mapping of TPNs and siRNA@TPNs (*n* = 3). G) The protective effect of TPNs against siRNA degradation after incubating with serum for different time periods (*n* = 3). Data were presented as mean ± SD.

The TPNs were synthesized via EGCG oxidation and then self‐polymerization to assemble nanoparticles according to our previous report (**Figure** **1**A).^[^ ^25^ ^]^ To test the potential of TPNs as gene vector, an siTNF‐*α* was used as proof‐of‐concept demonstration, which was added during TPNs preparation. To allow convenient observation, siRNA was labeled with a FAM fluorophore, through which the siRNA loading could be visualized by both fluorescent image and fluorescence intensity (Figure 1B). Compared to that of the siRNA/EGCG mixture, the fluorescence was almost vanished for the siRNA@TPNs complex due to fluorescence quenching effect of TPNs, indicating successful encapsulation of siRNA into TPNs structure. Interestingly, additional GSH treatment could rapidly recover the fluorescence to some extent, suggesting GSH‐mediated biodegradation of TPNs to trigger siRNA release. To confirm this, we further investigated the release profile of siRNA under different conditions (Figure S1, Supporting Information). In the absence of GSH, after initial release within 1 h (≈13%), a sustained release phase was observed over 24 h with cumulative release of ≈22%. In presence of GSH, by contrast, the release was markedly accelerated with cumulative release to be 44%. Such GSH‐responsive property is beneficial for intracellular delivery of siRNA, since GSH is abundant inside cells.^[^ ^26^ ^]^ The loading capacity was then measured by gel electrophoresis to quantify the unloaded siRNA (Figure 1C). TPNs could encapsulate ≈80% siRNA over the tested feeding concentrations (up to 12 µm), demonstrating an excellent loading capacity. After siRNA loading, siRNA@TPNs showed slight increase of particle size (Figure 1D), while the *ζ* potential became even more negative (Figure 1E).

To probe the structure, siRNA@TPNs was further characterized by TEM micro‐image (Figure 1F), which had a comparable morphology to that of TPNs, while the elemental mapping showed an obvious P signal within the nanoparticle, which was originated from siRNA loading. Next, the protective effect of the nanoparticles was studied by challenging siRNA@TPNs with serum (Figure 1G). From the gel band image, free siRNA was completely digested in 8 h due to its instability under physiological condition, which is one of its key limitations for in vivo applications. For siRNA@TPNs, by contrast, no degradation band was observed over 8 h, verifying effective siRNA encapsulation by nanoparticles to prevent its biological degradation.

### 3.2 Versatility of TPNs to Encapsulate Various Types of Nucleic Acids

**Figure 2:**
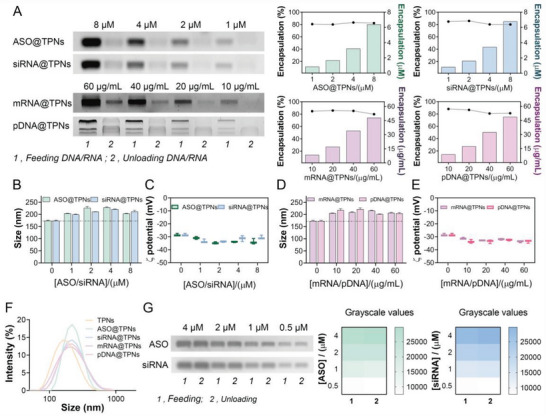
TPNs as a versatile gene vector to package various types of nuclei acids. A) Gel images and intensity quantification to show the loading capacity of TPNs toward ASO, siRNA, mRNA, and pDNA at different feeding concentrations. B,D) The particle size and C,E) *ζ* potential of ASO@TPN, siRNA@TPNs, mRNA@TPNs, and pDNA@TPNs prepared at different concentrations (*n* = 3). F) The size distribution of TPNs, ASO@TPNs, siRNA@TPNs, mRNA@TPNs, and pDNA@TPNs. G) The gel image and gray scale values to show the adsorption of ASO and siRNA on TPNs surface at different feeding concentrations (*n* = 3). Data were presented as mean ± SD.

Motivated by the excellent siRNA loading capacity, we then tested the versatility of TPNs as vector for various types of nucleic acids, including ASO, siRNA, mRNA, and pDNA. Likewise, the nucleic acids were added during TPNs preparation, and then characterized by gel electrophoresis, where ASO/siRNA (1–8 µm) were labeled by FAM fluorophore and mRNA/pDNA (10–60 µg mL^−1^) were stained by Goldview Nucleic Acid Gel Stain (**Figure** **2**A). Consistent with the above result, TPNs could effectively load all kinds of nucleic acids with efficiency of ≈80% over the tested concentrations, demonstrating the versatility for various gene deliveries. Slight particle size increase and surface charge decrease was also observed after nucleic acids loading (Figure 2B–F), while the resulting nanoparticles could maintain the particle size over 15 days (Figure S2, Supporting Information), indicating excellent loading stability. Notably, both TPNs and nuclei acids/TPNs complex displayed negative surface charge, which was different from the widely used cationic gene vectors. This can be ascribed to the distinct gene loading mechanism of the TPNs vector as illustrated in Scheme 1, in which EGCG was first intercalated into nucleic acids and its in situ oxidation/polymerization allowed gene encapsulation. As a control, TPNs were prepared first, and then mixed with ASO or siRNA. In this case, almost no nuclei acids were loaded based on the gel images, likely due to negatively charged nanoparticle surface to repel nucleic acids (Figure 2G).^[^ ^23^ ^]^ Therefore, nuclei acids were encapsulated into TPNs structure rather than adsorbed on particle surface.

### 3.3 A Comparison of TPNs and Cationic Materials as Gene Vector

**Figure 3:**
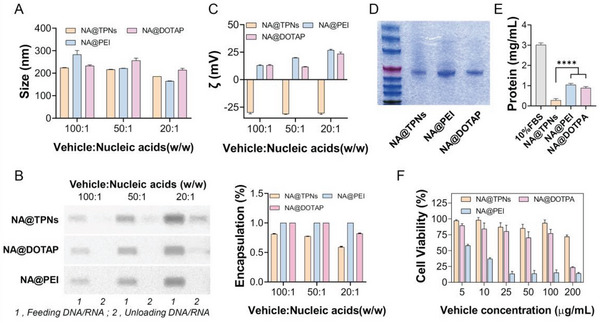
Comparison of TPNs with PEI and DOTAP gene vectors. A) Particle size, B) nucleic acids loading, and C) *ζ*‐potential of TPNs, PEI, and DOTAP after encapsulation of different masses of nucleic acids when the vehicle mass is kept constant (*n* = 3). D) Protein adsorption on different carriers examined by gel electrophoresis stained with Coomassie brilliant blue. E) Quantification of protein adsorption on different carriers by BCA (*n* = 3). F) The cytotoxicity of different carriers (*n* = 3). Data were presented as mean ± SD. Statistical comparisons were performed using one‐way ANOVA for (E). ****p < 0.0001.

Compared to the commonly used cationic gene vectors, TPNs employed a totally different mechanism for nucleic acids loading. To have a more detailed comparison of this difference, several characterizations were performed by using the representative cationic liposome DOTAP and cationic polymer PEI as controls. All these vectors could form nanoparticles upon mixing with nucleic acids (**Figure** **3**A), and effective nucleic acids loading was achieved based on gel image quantifications (Figure 3B). However, only TPNs showed negative charge while the other two had positive surface (Figure 3C). Note that the surface properties have strong influences on in vivo fate of nanoparticles. For example, various types of serum proteins could adsorb on cationic liposomes through electrostatic interactions to form a thick layer of protein corona, which in turn accelerates their clearance via mononuclear phagocytic system, and also affect the intracellular delivery.^[^ ^27^, ^28^, ^29^ ^]^ To test this, the vectors were incubated with serum, and the protein adsorption was quantified by gel electrophoresis and BCA protein kit (Figure 3D,E). Obviously, TPNs showed significantly lower level of protein corona than the positive vectors, which would benefit for its vivo circulation.

Another important concern of gene vectors is their biocompatibility, and we studied this parameter by MTT assay. The cationic PEI vector was highly cytotoxic because of its extremely positive charge to disrupt the cell membrane (Figure 3F). Likewise, the positively charged DOTPA also damaged cells at high concentration. On the other hand, most of the cells still remained alive upon treatment with both TPNs and TPNs/nucleic acids complex at various concentrations (Figure 3F and Figure S3, Supporting Information), and this result was further confirmed by live/dead cell double staining (Figure S4, Supporting Information). Therefore, TPNs were considered as a better gene vector for biomedical applications.

### 3.4 Intracellular Gene Delivery by TPNs to Promote Endosomal Escape and Gene Functionalization

**Figure 4:**
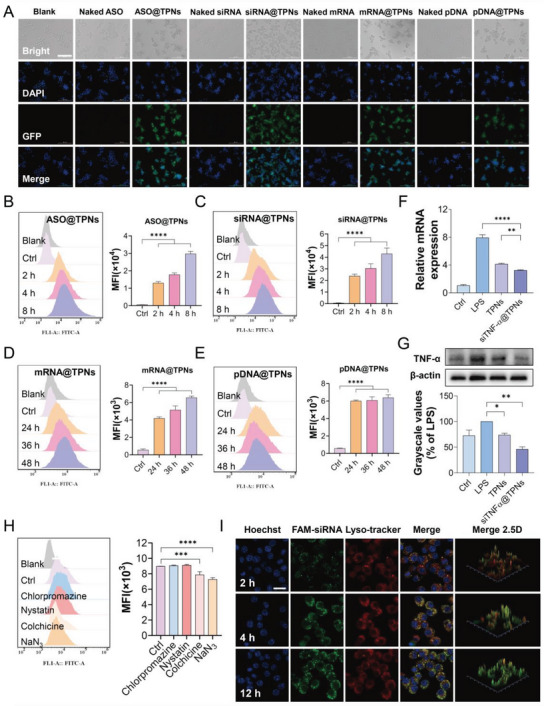
In vitro transfection of various nuclei acids by TPNs. A) The fluorescence images of RAW264.7 cells incubated with ASO/siRNA (8 h) or mRNA/pDNA (36 h) in the naked form or encapsulated by TPNs. Scale bar = 100 µm. The flow cytometry to quantify the cellular internalization of B) ASO@TPNs, C) siRNA@TPNs, D) mRNA@TPNs, and E) pDNA@TPNs after incubating for different timepoints (*n* = 3). F) The mRNA levels and G) protein levels of TNF‐*α* in RAW264.7 cells post different treatments (*n* = 3). H) The cellular internalization of siRNA@TPNs with pre‐treatment of various endocytosis inhibitors (*n* = 3). I) The intracellular co‐localization of siRNA@TPNs with lysosomes after incubating for different timepoints. Scale bar = 20 µm. Data were presented as mean ± SD. Statistical comparisons were performed using one‐way ANOVA for (B–H). *p < 0.05, **p < 0.01, ***p < 0.001, ****p < 0.0001.

Having demonstrated the gene loading capacity and biocompatibility, we next studied the capability of TPNs to facilitate intracellular delivery of the nucleic acids. To track the cell internalization, ASO and siRNA were labeled with a green fluorescent FAM fluorophore, while mRNA and pDNA were encoded with a GFP gene. In absence of transfection reagent, all naked nucleic acids showed minimal signal inside cells (**Figure** **4**A). Using TPNs as delivery vector, by contrast, strong fluorescence was observed for all of them. Notably, obvious signals from mRNA and pDNA groups were also observed, demonstrating their successful gene expression. We then quantified the fluorescence by flow cytometry, and specifically, ASO and siRNA showed gradually intensified signal up to 8 h, indicating a time‐dependent internalization (Figure 4B,C). For mRNA and pDNA, on the other hand, strong GFP intensity was detected after 24 h, and the signal could last at least for 48 h (Figure 4D,E).

While the function of mRNA and pDNA can be directly evidenced by GFP expression, the activity of ASO and siRNA should be studied by target gene suppression. To demonstrate this, the siTNF‐*α* was employed, and macrophages were stimulated with LPS to allow M1 polarization with upregulation of TNF‐*α* (Figure 4F,G). As expected, siTNF‐*α*@TPNs treatment could effectively knockdown TNF‐*α* at both mRNA and protein level, validating the efficacy of such gene delivery system. Interestingly, the TPNs alone could also suppress TNF‐*α* to some extent, which can be ascribed to its intrinsic anti‐inflammatory activity.^[^ ^25^ ^]^ Collectively, all these results manifested that functional nuclei acids could be delivered by TPNs into cells to exert their respective functions.

Next, the cellular uptake mechanism of the delivery system was explored by using various endocytosis inhibitors, including chlorpromazine, nystatin, colchicine, and NaN_3_, to prevent clathrin‐, caveolin‐, and macropinocytosis‐mediated endocytosis or energy, respectively. For these treatments, NaN_3_ had the most significant influence, suggesting energy‐dependent nanoparticle internalization. Besides, colchicine could also decrease the fluorescence signal, indicating the critical role of micropinocytosis‐mediated cellular uptake (Figure 4H). It is known that macropinocytosis proceeds from the cell membrane to form macropinosome and then sort the cargos through endosomes.^[^ ^30^ ^]^ Thus, the endosomal escape is a crucial step for effective gene delivery. Given the effective mRNA/pDNA expression and siRNA‐mediated gene downregulation, we speculated that TPNs could promote endosomal escape of the nucleic acids. To confirm this, the lysosomes were stained with LysoTracker Red and their co‐localization with the internalized siRNA was observed (Figure 4I). After 2 h incubation, the siRNA was mainly delivered in endo/lysosomes to produce merged orange fluorescence, consistent with the mechanism of micropinocytosis‐mediated internalization. With prolonged incubation, the green fluorescence gradually enhanced due to more nanoparticle internalization, but the signal progressively separated from red fluorescence. This result verified the effective endosomal escape of the nanoparticles, and the underlined mechanism might be attributable to the surface abundant phenolic hydroxyl groups in TPNs with pH‐buffering capacity to endow the “proton‐sponge effect”.^[^ ^31^ ^]^

### 3.5 Intrinsic Biofunctions of the TPNs Vector

In our study, EGCG, a natural polyphenol, was employed as raw material to prepare TPNs. EGCG has been reported with broad pharmacological functions owing to its anti‐inflammatory and anti‐oxidant activities.^[^ ^32^ ^]^ We recently reported that EGCG could oxidize into polymers and then assemble into TPNs, and the obtained TPNs possess robust RONS scavenging activities.^[^ ^25^ ^]^ RONS is known to play deleterious role in various diseases, and scavenging RONS is an important strategy to manage these diseases.^[^ ^10^ ^]^ As such, besides serving as a versatile gene vector, we were also intrigued whether TPNs would retain the RONS scavenging activities after nucleic acids loading to synergize the gene therapy. To test this, various radical species were added or in situ generated in test tube, and the RONS scavenging activities of siRNA@TPNs were explored one by one. As expected, TPNs had good radical scavenging ability at the test tube level toward various types of RONS (Figure S5, Supporting Information).

**Figure 5:**
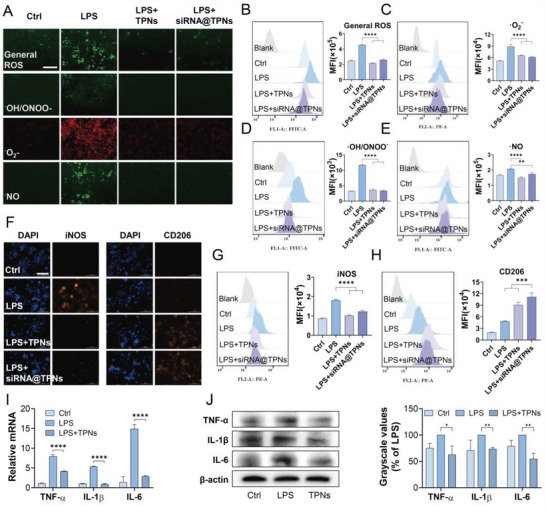
Antioxidation, anti‐inflammation, and macrophage repolarization effects of TPNs vector. A) The fluorescence images of RAW264.7 cells with different treatments showing the capacity of siRNA@TPNs to scavenge RONS. Scale bar = 100 µm. The flow cytometry results of TPNs to scavenge B) general ROS, C) ·O_2_^−^, D) ·OH/ONOO^−^, and E) ·NO (*n* = 3). F) Immunofluorescence images of iNOS and CD206 in RAW264.7 cells with different treatments. Scale bar = 100 µm. The flow cytometry results of G) iNOS and H) CD206 expression in RAW264.7 cells with different treatments (*n* = 3). I) The mRNA levels and J) protein levels of TNF‐*α*, IL‐1*β*, and IL‐6 in RAW264.7 cells post different treatments (*n* = 3). Data were presented as mean ± SD. Statistical comparisons were performed using one‐way ANOVA for (B–E) and (G–J). *p < 0.05, **p < 0.01, ***p < 0.001, ****p < 0.0001.

Given the above results, we further studied the biofunctions of siRNA@TPNs at cellular level by using RAW264.7 cells, in which the cells were pre‐treated with LPS to induce oxidative stress with RONS overproduction inside cells. Using different indicators, the RONS species, including the general ROS, ·OH/ONOO^−^, ·O_2_^−^, and ·NO, can be visualized by their respective fluorescence signal (**Figure** **5**A). All these radicals were overproduced upon LPS treatment, while the fluorescence signals decreased to almost background level after TPNs and siRNA@TPNs treatments, demonstrating the retained RONS scavenging activity of siRNA@TPNs as gene vector. We then quantified the fluorescence by flow cytometry, and consistently, both TPNs and siRNA@TPNs could significantly reduce the fluorescence intensity for all types of the tested RONS (Figure 5B–E).

Stimulated by the RONS with oxidative stress state, macrophages tend to polarize into pro‐inflammatory M1 phenotype, which has been found to play paramount roles in occurrence and development of various inflammatory diseases.^[^ ^33^, ^34^, ^35^ ^]^ Therefore, reprogramming the pro‐inflammatory M1 macrophages into anti‐inflammatory M2 macrophages has become a promising diseases treatment modality. Interestingly, we and other research groups have shown that scavenging the intracellular RONS is an effective method to promote M1‐to‐M2 macrophage repolarization.^[^ ^10^, ^36^ ^]^ Inspired by this, the macrophage regulating activity of siRNA@TPNs was also studied. The phenotype of the macrophage was identified by immunofluorescent staining of iNOS (M1 marker) and CD206 (M2 marker) (Figure 5F). The iNOS signal markedly increased upon LPS stimulation, indicating M1 polarization. With additional TPNs or siRNA@TPNs treatment, iNOS fluorescence was strongly weakened accompanied by the increase of CD206 signal, suggesting M1‐to‐M2 repolarization. We further quantified the intensity, and consistent results were obtained for all treatments (Figure 5G,H). In line with M2 macrophage repolarization, several typical pro‐inflammatory cytokines, including TNF‐*α*, IL‐1*β*, and IL‐6, were also suppressed by the nanoparticles (Figure 5I,J). Therefore, TPNs presents as a type of multi‐functional nanomaterial, which could not only serve as a versatile gene vector to load various types of nucleic acids, but also exert intrinsic activities to scavenge RONS, promote M1‐to‐M2 macrophages repolarization, and suppress inflammation.

### 3.6 Liver Accumulation of siRNA@TPNs for Hepatitis Therapy

**Figure 6:**
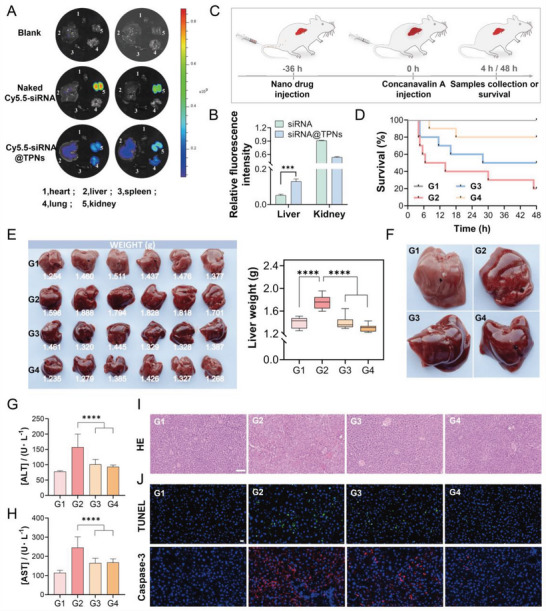
In vivo delivery of siRNA by TPNs for the treatment of Con A‐induced acute hepatitis. A) Distribution and B) fluorescence quantification of siRNA in major organs of mice at 24 h post injection with PBS, naked Cy5.5‐siRNA, or Cy5.5‐siRNA@TPNs (*n* = 3). C) Schematic illustration of in vivo experiments. D) Survival curves of mice post different treatments (*n* = 10). E) The photographs and weight of livers in mice post different treatments (*n* = 6). F) Photographs of specific livers enlarged from (E). The levels of G) ALT and H) AST in serum after various treatments (*n* = 6). I) H&E staining images of livers after different treatments. Scale bar = 100 µm. J) TUNEL and caspase‐3 immunofluorescence staining images of livers after various treatments. Scale bar = 20 µm. G1, PBS; G2, Con A; G3, Con A+TPNs; G4, Con A+siRNA@TPNs. Data were presented as mean ± SD. Statistical comparisons were performed using Student's t‐test for (B) and one‐way ANOVA for (E) and (G–H). ***p < 0.001, ****p < 0.0001.

To explore the real application of such TPNs‐based gene vector, several in vivo experiments were performed. We first tested the hemocompatibility of the nanoparticles, while almost no hemolysis was observed with concentration up to 320 µg mL^−1^ (Figure S6, Supporting Information). Therefore, the nanoparticles can be directly administrated via intravenous injection. Then, the biodistribution was investigated by encapsulating a Cy5.5‐labeled siRNA into TPNs to enable in vivo fluorescent imaging. The naked siRNA showed high fluorescence signal in kidneys at 24 h post‐injection (**Figure** **6**A), indicating renal clearance of the nucleic acids. For siRNA@TPNs group, by contrast, strong fluorescence was also noticed in liver, attributable to the liver entrapment of the nanoparticles.^[^ ^37^ ^]^ The fluorescence was then quantified for direct comparison, in which siRNA@TPNs exhibited 2.5‐fold higher intensity in liver than naked siRNA (Figure 6B), confirming the liver targetability of the nanoparticles.

Given these results, we speculated that siRNA@TPNs were particularly suitable for the treatment of liver‐related diseases. Taking the intrinsic biofunctions of the TPNs vector into consideration, we decided to attempt the use of siRNA@TPNs to treat acute hepatitis. Acute hepatitis is a disease in which liver cells are damaged and liver function is impaired due to a variety of pathogenic factors, typically alcoholic hepatitis and viral hepatitis. It is pathologically characterized by extensive hepatocellular oedema and liver stasis or apoptosis caused by massive immune cell infiltration, RONS production, and inflammatory cytokine secretion. TPNs alone might have some therapeutic effects for hepatitis by virtue of RONS scavenging and anti‐inflammatory activities, and the loaded siRNA was designed to silence caspase‐3 (Figure S7, Supporting Information). Through this design, TPNs and the payload siRNA were expected to synergize with each other for better therapy.

Con A was injected into mice to build hepatitis animal model,^[^ ^38^, ^39^ ^]^ and the experimental procedure was illustrated in Figure 6C. The injected Con A could accumulate in liver to activate the immune system and induce T‐cell‐dependent liver damage.^[^ ^40^, ^41^ ^]^ As a result, Con A‐induced hepatitis resulted in quick mice death within 6 h, and 48 h survival rate was only 20%. Upon treatment with TPNs, an obvious increase of mice survival was observed, confirming the protective effect of RONS scavenging nanomaterials against hepatitis damage.^[^ ^42^ ^]^ Notably, the siRNA@TPNs group showed an even better efficacy based on mice survival curve, demonstrating the synergistic effect of TPNs and its payload siRNA (Figure 6D). For more detailed evaluation, the liver samples were collected, and the weight was recorded (Figure 6E). For the model mice with Con A treatment, the liver weight increased significantly, and this could be clearly seen from the enlarged images (Figure 6F). This symptom was due to congestion of the liver as well as edema of the hepatocytes. With TPNs and siRNA@TPNs therapies, by contrast, the liver weight remained unchanged, confirming the protection effect. In addition, the liver injury was further characterized by the typical indicators of ALT and AST (Figure 6G,H). While the model group showed significant increase, both indexes were within the normal range after TPNs and siRNA@TPNs treatments.

To confirm this therapeutic efficacy, the liver samples were further evaluated by histological analysis. Based on the H&E staining, the liver of model group with Con A treatment showed blurred lobular structure, overall aqueous degeneration, focal hepatocellular steatosis, stasis and dilatation of the hepatic blood sinusoids, and interstitial vessels (Figure 6I). However, siRNA@TPNs treatment could strongly alleviate all these pathological changes. Overall, the level of liver tissue damage was in the range of Con A > Con A+TPNs > Con A+siRNA@TPNs. To verify the efficacy of siRNA, TUNEL staining and caspase‐3 immunofluorescence staining were further performed (Figure 6J), in which the weakest fluorescence was observed for siRNA@TPNs group. Therefore, siRNA@TPNs could effectively accumulate into liver and silence caspase‐3 to inhibit the apoptosis of hepatocytes, which in turn synergize with RONS scavenging activity of TPNs to realize liver protection.

### 3.7 Biofunctions of TPNs Vector to Scavenge RONS and Inhibit Inflammation for Enhanced Hepatitis Therapy In Vivo

**Figure 7:**
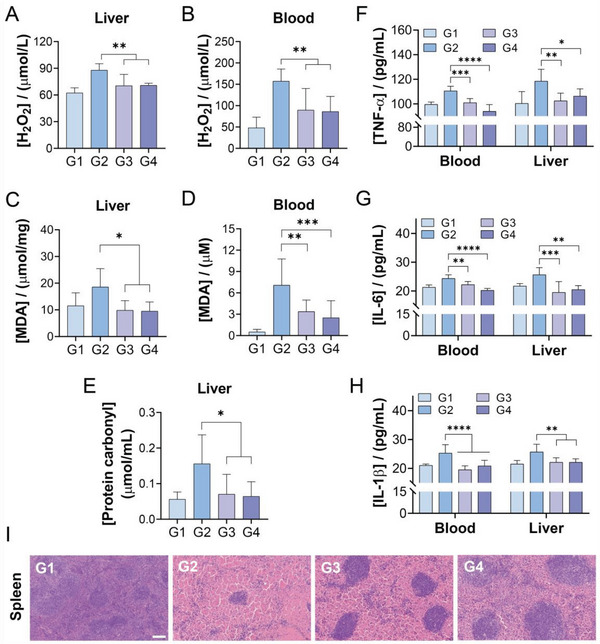
In vivo anti‐oxidant and anti‐inflammatory activities of the gene vector. H_2_O_2_ concentration in A) liver and B) blood of mice in each group (*n* = 6). MDA concentration in C) liver and D) blood of mice in each group (*n* = 6). E) The protein carbonyl content in the liver of mice with different treatments (*n* = 6). The levels of inflammatory cytokines including F) TNF‐*α*, G) IL‐6, and H) IL‐1*β* in the liver and blood of mice after various treatments (*n* = 6). I) H&E staining images of the spleen of mice with different treatments. Scale bar = 100 µm. G1, PBS; G2, Con A; G3, Con A+TPNs; G4, Con A+siRNA@TPNs. Data were presented as mean ± SD. Statistical comparisons were performed using one‐way ANOVA for (A–E) and two‐way ANOVA for (F–H). *p < 0.05, **p < 0.01, ***p < 0.001, ****p < 0.0001.

Compared to most other gene vector, the unique advantage of TPNs is its intrinsic biofunctions, which could synergize the treatment efficacy of inflammatory diseases such hepatitis. For example, hepatitis model mice showed high H_2_O_2_ concentration in both liver tissues and blood due to oxidative stress (**Figure** **7**A,B), accompanied by the increase of MDA and protein carbonyl levels (Figure 7C–E), all of which have deleterious effects on liver. Notably, TPNs and siRNA@TPNs could recover all these parameters to normal level, attributable to their activity to eliminate RONS. Moreover, the anti‐inflammatory effect was also studied by measuring the cytokines. As expected, the nanoparticles could effectively alleviate hepatitis‐induced cytokine storm, resulting in significant decrease of cytokines including TNF‐*α*, IL‐6, and IL‐1*β* (Figure 7F–H). To further examine the degree of inflammation, spleen was obtained for H&E staining (Figure 7I). The spleen of model mice showed subperitoneal oedema with an indistinct red medullary structure, marked bruising and haemorrhagic changes in the interstitium, and a significantly reduced white medullary area with an extremely irregular distribution. After nanoparticles treatment, by contrast, the basic structure of the spleen was unchanged, and the stasis was significantly improved compared to the Con A group, further demonstrating the anti‐inflammatory abilities of the nano‐vector.

In addition, the H&E staining was also performed on other major organs, including heart, lung, and kidneys (Figure S8, Supporting Information). Heart tissues was unaffected by Con A administration, while noticeable lung and kidney damages were seen, although these injuries were non‐organic. Specifically, the alveolar wall capillaries were dilated and congested, and the focal alveoli showed compensatory emphysematous changes. The interstitial vessels were dilated and congested, and inflammatory cell aggregates were seen in the lumen. The glomerular capillaries were congested, with a little exudated in the focal glomerular capsule and some crescent‐like structures, diffuse hydropic degeneration of the renal proximal tubules, and dilated and congested interstitial vessels. Fortunately, the nano‐vector could effectively protect all these organs with significantly alleviated symptoms. Moreover, the representative blood biochemical parameters including BUN and Cre were measured after treatments (Figure S9, Supporting Information), both of which were within the normal range. Collectively, siRNA@TPNs showed broad protective effect toward various organs especially liver. Together with its intrinsic biofunctions and high biocompatibility, such gene vector shows great potential for biomedical applications to treat various types of inflammatory diseases.

## 4 Conclusions

In summary, this work developed a simple yet versatile gene vector based on Mn^2+^‐catalyzed EGCG oxidation and polymerization, through which various types of nucleic acids could be effectively loaded with uniformed nano‐structures. TPNs showed obvious advantages over typical cationic vectors in terms of protein corona formation and biocompatibility, highlighting its suitability for in vivo applications. At cellular level, we have demonstrated the capability of TPNs to deliver ASO, siRNA, mRNA, and pDNA for intracellular functions, in which the internalization was mainly mediated by micropinocytosis with effective endo/lysosome escape. Notably, TPNs possessed robust RONS scavenging and anti‐inflammatory activities to promote macrophage M1‐to‐M2 phenotype polarization. Given such intrinsic bioactivities and liver targetability, TPNs vector was employed to deliver an sicaspase‐3 for hepatitis therapy, and a combinatorial therapeutic effect was achieved between payload and the cargo with significantly improved animal survival and liver damage protection. Integrating the properties of versatile gene loading, bioactivities, and excellent biocompatibility, such TPNs‐based vector would find broad biomedical applications.

## Conflict of Interest

The authors declare no conflict of interest.

## Supporting information

Supporting Information

## Data Availability

The data that support the findings of this study are available from the corresponding author upon reasonable request.
