# Supplementary material for: A General and Efficient Strategy for Gene Delivery Based on Tea Polyphenols Intercalation and Self‐Polymerization
Source: Adv Sci (Weinh). 2023 Jun 22;10(24):2302620. doi: 10.1002/advs.202302620 (PMC10460882; doi:10.1002/advs.202302620)
Supplement: Supplementary file 1 — Supporting Information [file ADVS-10-2302620-s001.pdf]

ADVANCED SCIENCE
Open Access

Supporting Information

for *Adv. Sci.*, DOI 10.1002/advs.202302620

A General and Efficient Strategy for Gene Delivery Based on Tea Polyphenols Intercalation and Self-Polymerization

Hao Chen, Lina Guo, Jinsong Ding, Wenhu Zhou and Yan Qi**

Supporting Information

A General and Efficient Strategy for Gene Delivery Based on Tea Polyphenols Intercalation and Self-polymerization

Hao Chen[1,3+], Lina Guo[2+], Jinsong Ding[2], Wenhu Zhou*[2] and Yan Qi*[1,3]

1. Department of Pathology, Zhanjiang Central Hospital, Guangdong Medical University, Zhanjiang, Guangdong, 524000, China

2. Xiangya School of Pharmaceutical Sciences, Central South University, Changsha, Hunan, 410013, China

3. Department of Pathology, Shihezi University School of Medicine & the First Affiliated Hospital to Shihezi University School of Medicine, Shihezi 832002, Xinjiang, China.

[+] These authors contributed equally to this work.

E-mail: zhouwenhuyaoji@163.com, qiyanyan-1998@163.com

Table S1. The sequences of the primer used in RT-PCR.

Primer	Forward (5'-3')	Reverse (5'-3')
β-actin	CTACAATGAGCTGCGTGTGG	CAGGTCCAGACGCAGGATGGC
Caspase-3	CGGGGTACGGAGCTGGACTGT	AATTCCGTTGCCACCTTCCTGTT
TNF-α	CTCATGCACCACCATCAAGG	ACCTGACCACTCTCCCTTTG
IL-1β	ATGAAGGGCTGCTTCCAAAC	TCTCCACAGCCACAATGAGT
IL-6	GGAGCCCACCAAGAACGATA	ACCAGCATCAGTCCCAAGAA

Table S2. The sequences of the siRNA used in this study.

Gene	Sense(5'-3')	Antisense(5'-3')
NC	UUC UCC GAA CGU GUC ACG UTT	ACG UGA CAC GUU CGG AGA ATT
sicaspase-3 1	GGG AUC UAU CUG GAC AGU ATT	UAC UGU CCA GAU AGA UCC CTT
sicaspase-3 2	GCC AAC CUC AGA GAG ACA UTT	AUG UCU CUC UGA GGU UGG CTT
sicaspase-3 3	GGA UAG UGU UUC UAA GGA ATT	UUC CUU AGA AAC ACU AUC CTT
siTNF-α	GAC AAC CAA CUA GUG GUG CTT	GCA CCA CUA GUU GGU UGU CTT

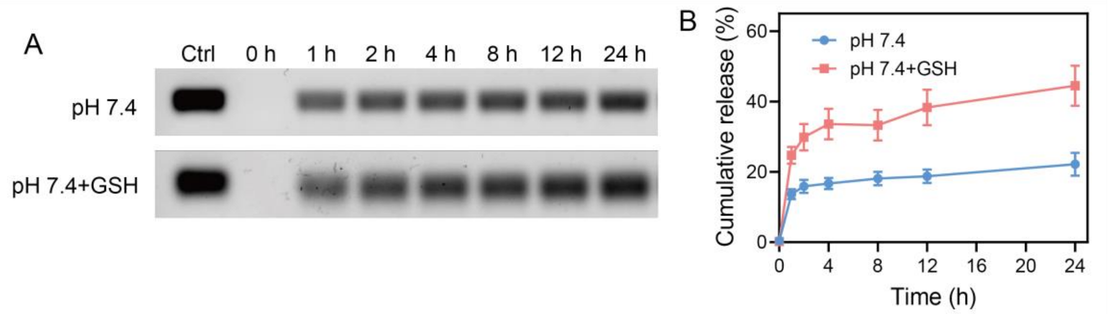

Figure S1. (A) The gel electrophoresis showing the release of siRNA from TPNs at different time points in presence or absence of GSH. (B) The cumulative release percentage of siRNA determined from (A). The siRNA was labelled with a FAM fluorophore to allow convenient tracking on gel electrophoresis.

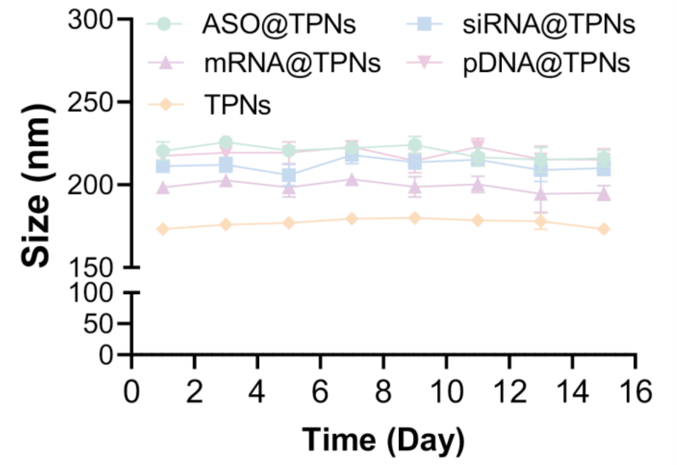

Figure S2. The size stability of TPNs, ASO@TPNs, siRNA@TPNs, mRNA@TPNs and pDNA@TPNs within 15 d.

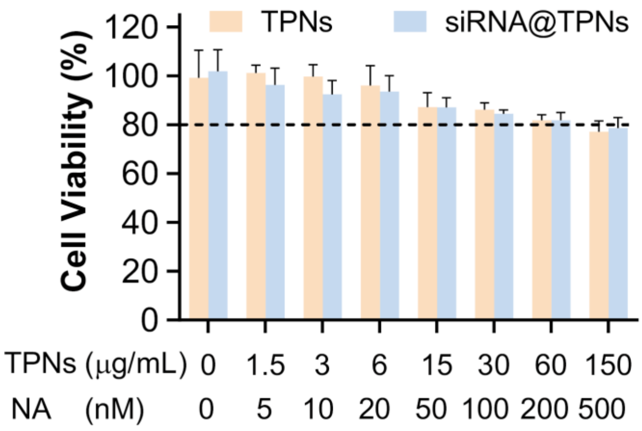

Figure S3. The cell viability of RAW264.7 cells post treatment with different concentrations of TPNs or siRNA@TPNs.

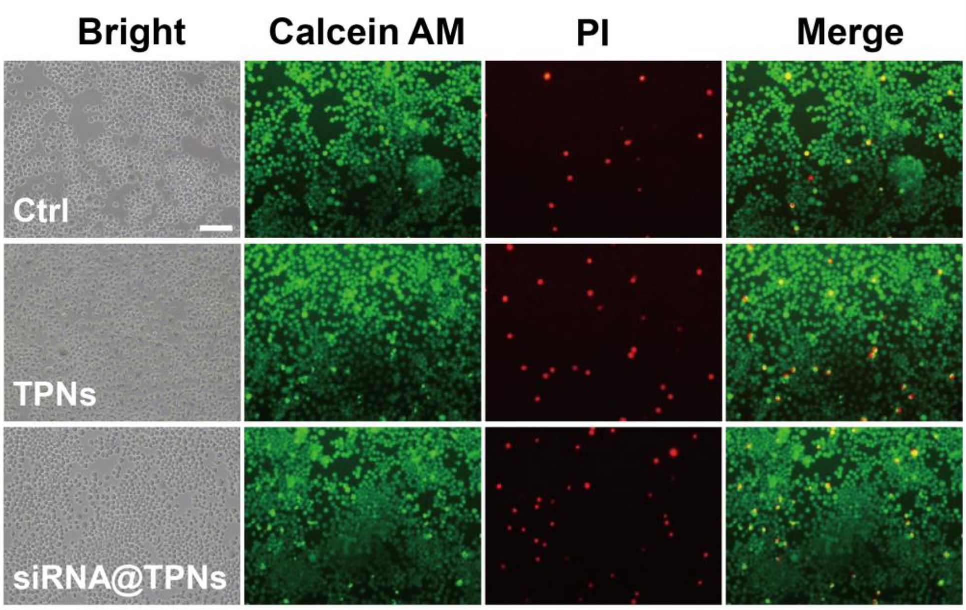

Figure S4. The live/dead staining of RAW264.7 cells post different treatments. Scale bar = 100 μm.

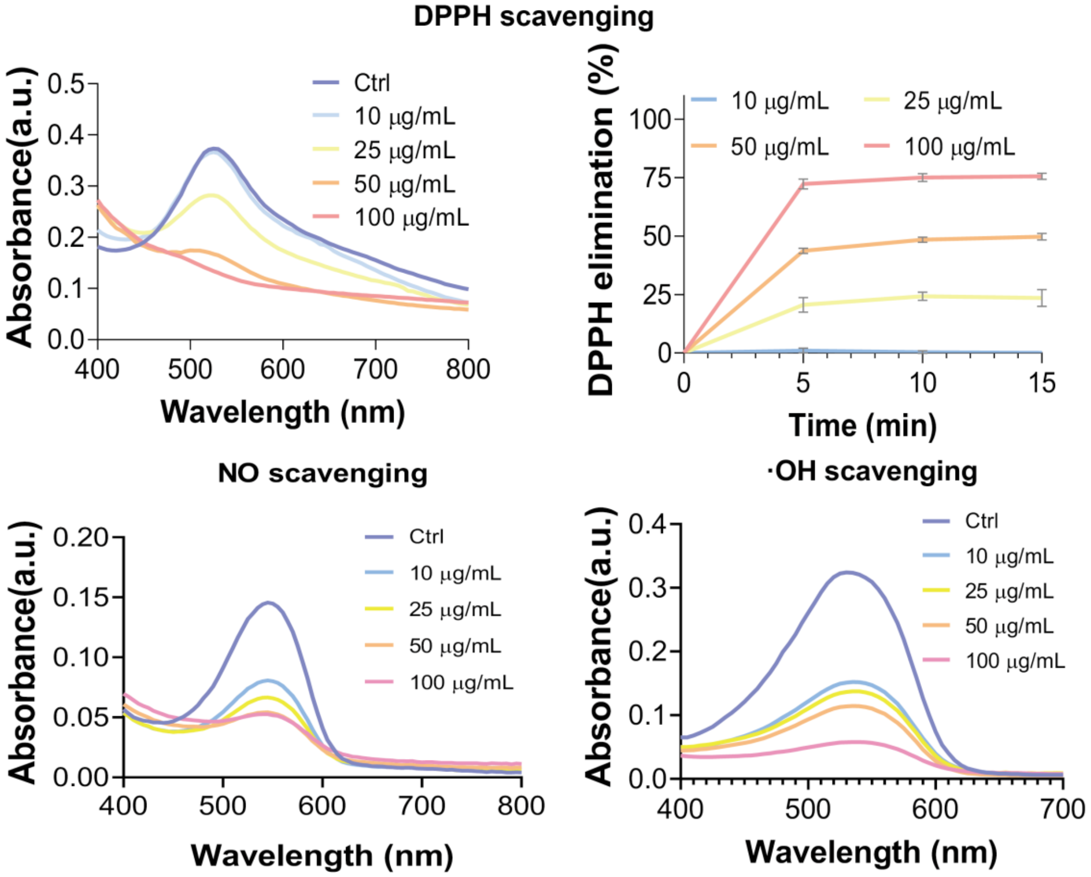

Figure S5. The RONS scavenging activity of TPNs at different concentrations detected by UV-Vis.

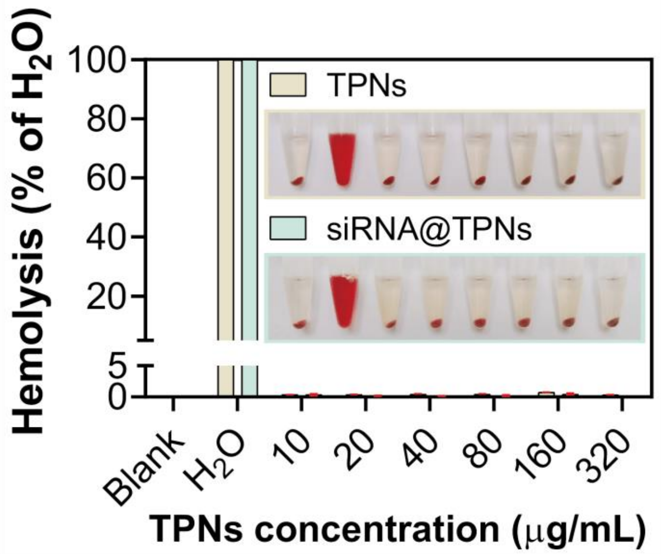

Figure S6. The hemolysis rate of TPNs and siRNA@TPNs at different concentrations.

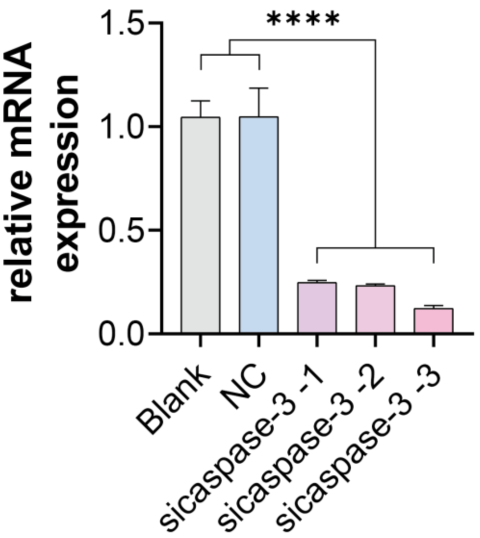

Figure S7. The relative mRNA levels of caspase-3 in RAW264.7 cells post different treatments.

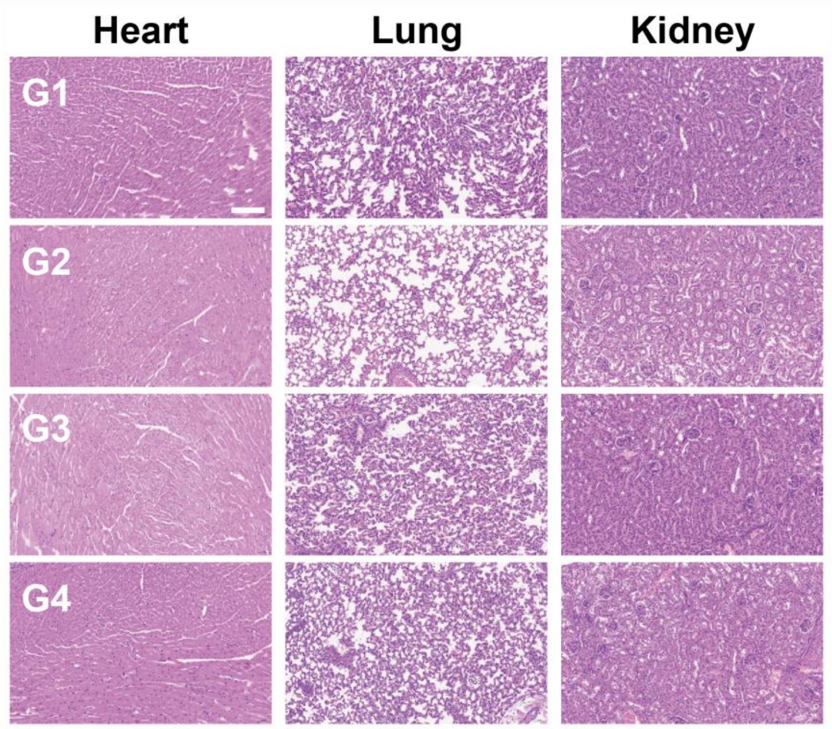

Figure S8. The H&E staining images of heart, lung and kidneys of the mice post different treatments. G1, PBS; G2, Con A; G3, Con A+TPNs; G4, Con A+siRNA@TPNs. Scale bar = 100 μm.

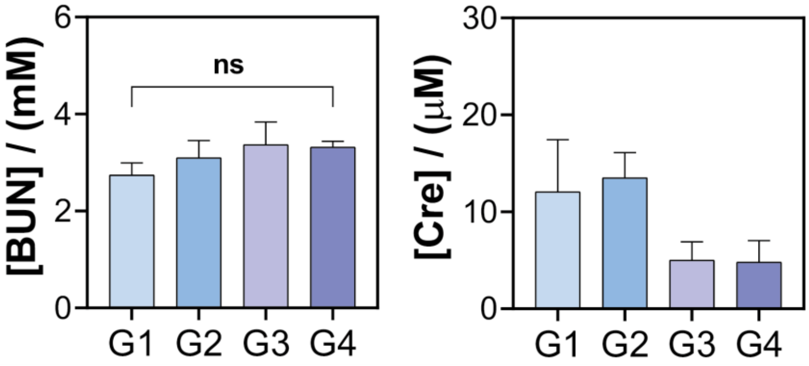

Figure S9. The serum levels of BUN and Cre in mice post different treatments. G1, PBS; G2, Con A; G3, Con A+TPNs; G4, Con A+siRNA@TPNs.
